# How I do it: Modified operation technique for MR Conditional OSIA 3: anterior based flap for covering the implant

**DOI:** 10.1007/s00405-025-09202-5

**Published:** 2025-01-19

**Authors:** Ioana T. Brill, Stefan Dazert

**Affiliations:** Department of Otorhinolaryngology, Head and Neck Surgery, Ruhr Universitiy Bochum, St. Elisabeth-Hospital, Bochum, Germany

**Keywords:** OSIA3, Bone conductive hearing aids, BI300, MRI

## Abstract

**Background:**

The new OSIA3 is 1.5 T and 3 T MR-Conditional. Skin related issues are the most common complications. Performing MRI, the implant is exposed to magnetic torque forces, thus creating more tension over the incision lines.

**Methods:**

We present a modified surgical technique for implanting OSI300 step-by-step. We prefer an anterior based muscle-periosteal flap to cover the implant as an integral part of the procedure.

**Conclusion:**

The anterior based muscle-periosteal flap and performing a 3-layer-suture appears to be a safe method that does not permit skin-tension directly over the implant and may help preventing implant extrusion especially with the new OSI300.

## Introduction

Some active transcutaneous bone conduction hearing implants on the market—BoneBridge (MED-EL GmbH, Innsbruck, Austria), OSIA 2 (Cochlear Ltd., Sydney, Australia), Sentio (Oticon, Smørun, Dänemark)—are MRI conditional at 1.5 Tesla with head bandage. The OSIA 3 System (Cochlear Ltd., Sydney, Australia) is the first that allows MRI investigation at 3 T with magnet in place and no head bandage [1]. It is indicated for conductive and mixed hearing loss or as contralateral routing of signal (CROS) device in single sided deaf persons [2].

**Fig. 1:**
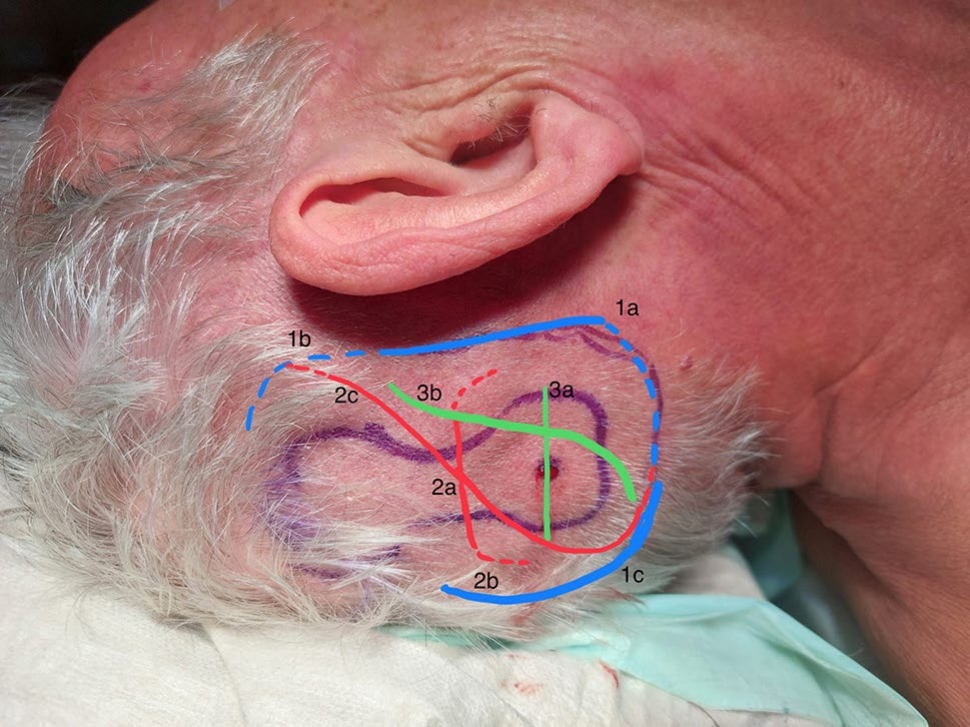
Different incision types (according to literature): 1 (blue): incisions as proposed by the manufacturer: 1a: anterior extended inferior (J-incision) – we perfom this incision as well as described in Methods step b.; 1b: anterior extended superior, 1c: posterior (C-shaped) skin incisions. 2 (red): modified incisions over the waist of the implant: 2a: straight; 2b: inverted U; 2c: curvilinear Sheffield-S incision. 3 (green): modified incisions across the site of the implant screw: 3a straight; 3b: curvilinear

Anterior extended inferior (J-incision) or superior, and posterior (C-shaped) skin incisions have been proposed by the manufacturer [2] and are performed by many surgeons [3, 4]. Modified incisions: over the waist of the implant either straight [5], inverted U [7] or curvilinear *Sheffield-S* incision [5], combined with an anterior one [6]; linear incisions across the site of the implant screw [5, 7, 8] have also been reported. The most commonly observed complications are skin related such as wound breakdown, skin infection [9] and partial implant extrusion at the incision site [10]. The most adopted incision, the anterior inferior extended one (J-incision), is the one also related to the most significant skin tension at the inferior edge of the actuator [7] (Fig. 1). We propose a modified technique that allows repositioning the implant site intraoperatively under direct view and avoids placing sutures and tension above the implant edge.

## Methods

Institutional review board approval and patients’ written consent were obtained.

### Description of the technique (video)

#### Operation planning

In our department, the operation usually is performed under general anesthesia. In preparation for the surgery, a CT-scan with measurement of the bone thickness and implant site planning as well as BAHA-headband trial and audiometry tests are performed.

A template is used to outline the implant on the skin at the desired site. The incision is planned 10–15 mm away from the device. Ultracain local anaestethic with adrenalin is injected into the surgical site. Single shot antibiotics are given only perioperatively.

#### Retroauricular flap J-shaped incision inferior extended at the hair line

We adopted the retroauricular J-incision anterior to the implant position at the hair line. The first incision cuts through skin und underlying fibrous layers, leaving the temporalis muscle and the posterior auricular muscle intact. This layer is followed posteriorly as far as the posterior implant border.

#### Relevant surgical anatomy: anteriorly based muscle periosteal flap (Fig. 2)

**Fig. 2:**
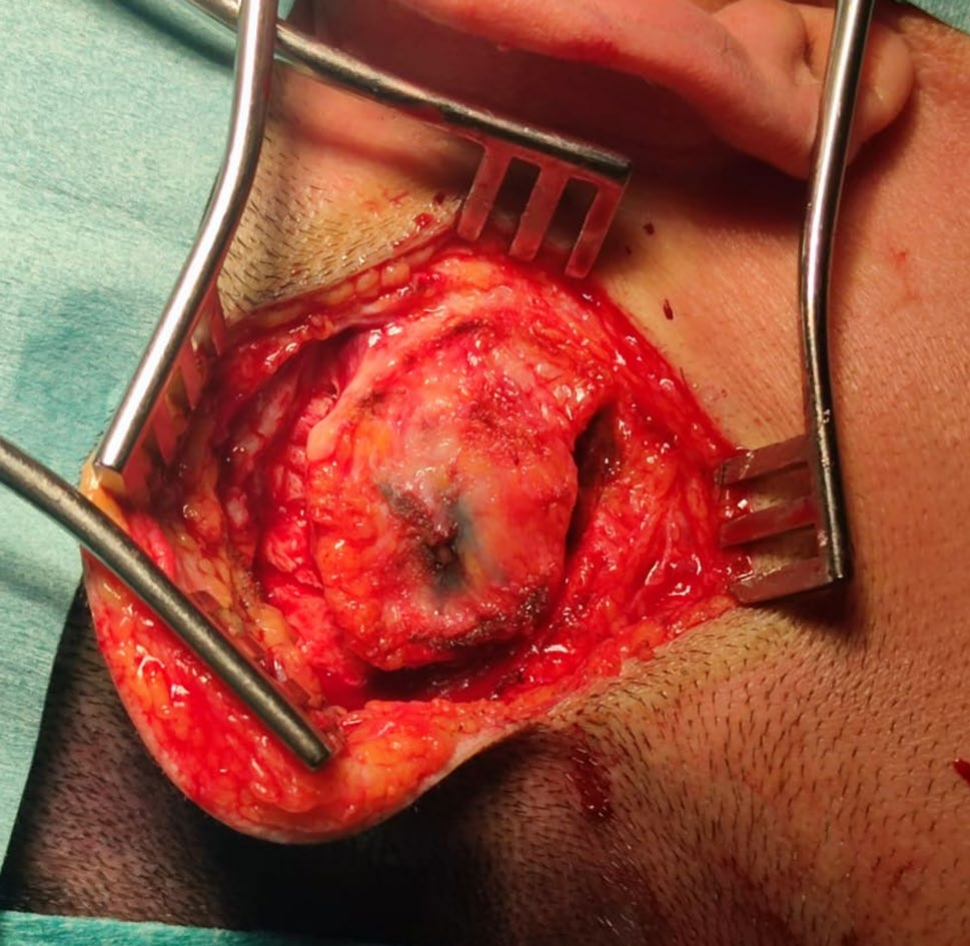
Anteriorly based muscle periosteal flap

The next incision is far posterior, at the planned posterior implant border up to the scull bone, cutting through the muscular and periosteal layer. Two more incisions are cut: superiorly at the inferior border of the temporalis muscle, and inferiorly at the inferior border of the implant site. Further dissection with bone contact anteriorly is widely exposing the bone for implant placement. Thus, an anterior based muscular periosteal flap is created.

We propose this flap for two reasons. Firstly, it allows more anterior repositioning of the implant if necessary, and secondly, no sutures will be placed above the implant.

#### Implant placement (Fig. 3)

**Fig. 3:**
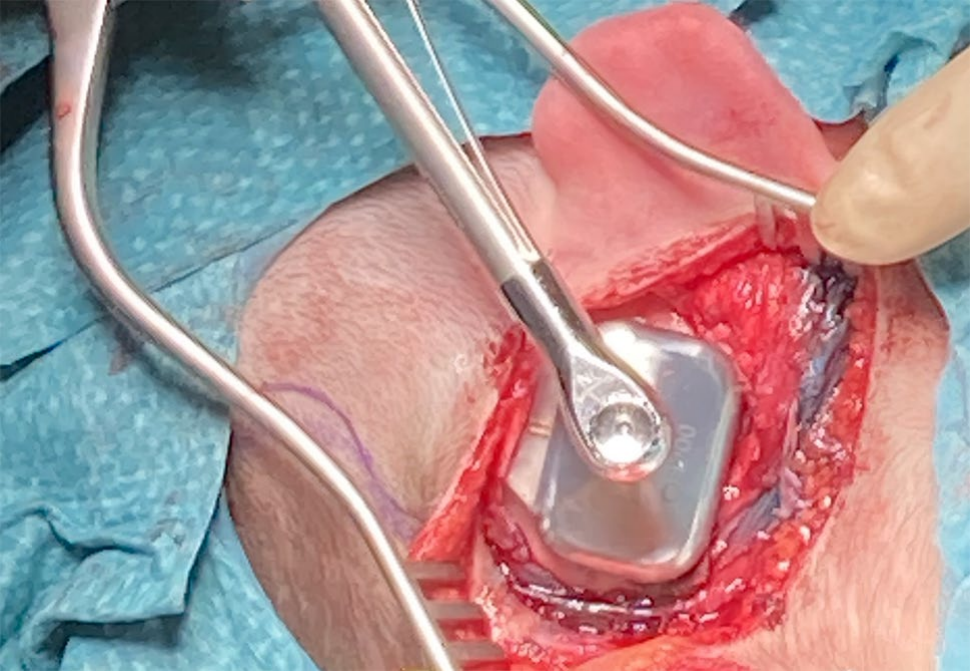
Implant placement

A flat part of the skull is identified for the placement of the actuator. With the anterior based flap described above, we are able to safely move the position of the implant a little bit anterior if necessary despite reducing the distance to the skin incision, not having to fear skin issues at a later point.

It is necessary to start drilling perpendicularly using the guide with the conical 3 mm guide drill with spacer at 2000 RPM. The initial conical drilling site is enlarged with the suited widening drill.

Using the guiding drill indicator, the implant is inserted at a perpendicular angle to the bone, applying torque forces of 40–50 Ncm for normal compact bone. To avoid bone heating and osteonecrosis at the implant site, constant and abundant irrigation is important.

Before applying the OSI300, the clearance indicator mounted on the BI300 is used to check the smooth, flat bony region. In patients with more edged skull surface and curved mastoid tips, we place the device more superiorly such, that the screw fixation site is 1–2 cm above the level of the external auditory canal. A less cosmetic bump with skin tension over the inferior implant edge can be prevented by this procedure that is also described by Deep et al. [7]. If the temporal line or temporoparietal sutures are prominent, polishing the bone with a diamond drill is helpful to ensure a flat surface and flush contour with the device in place. After the BI implant is positioned, the vibrating part OSI300 is fixed and thightened with a torque key at 25 Ncm. The actuator is flat, rigid and quite big (31.4 × 22 × 4.9 mm). If a bony bump cannot be avoided, a recess well like described by Arndt et al. [7] should be performed.

#### Closure (Fig. 4) and postoperative care

**Fig. 4:**
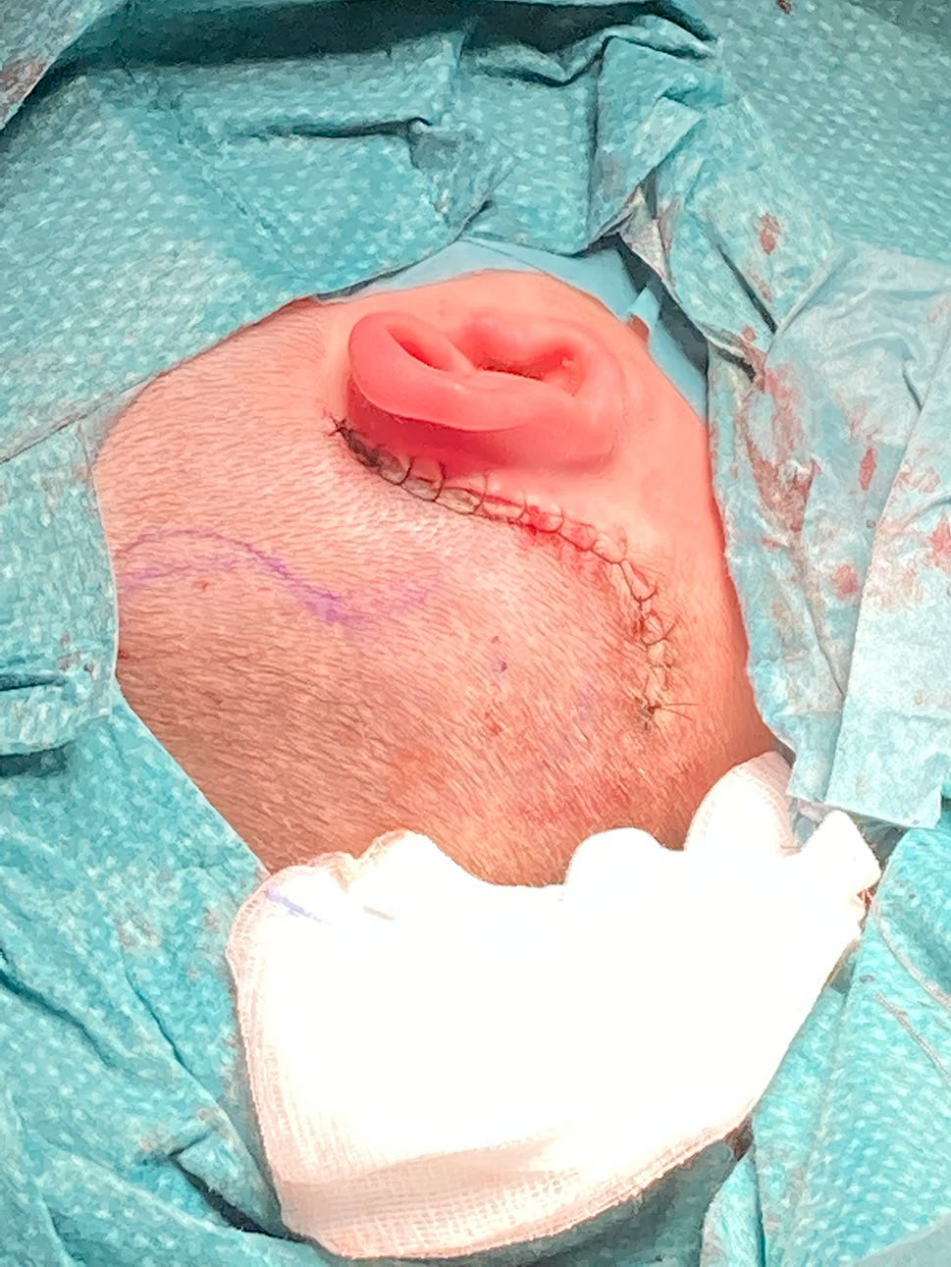
Wound suture

**Fig. 5:**
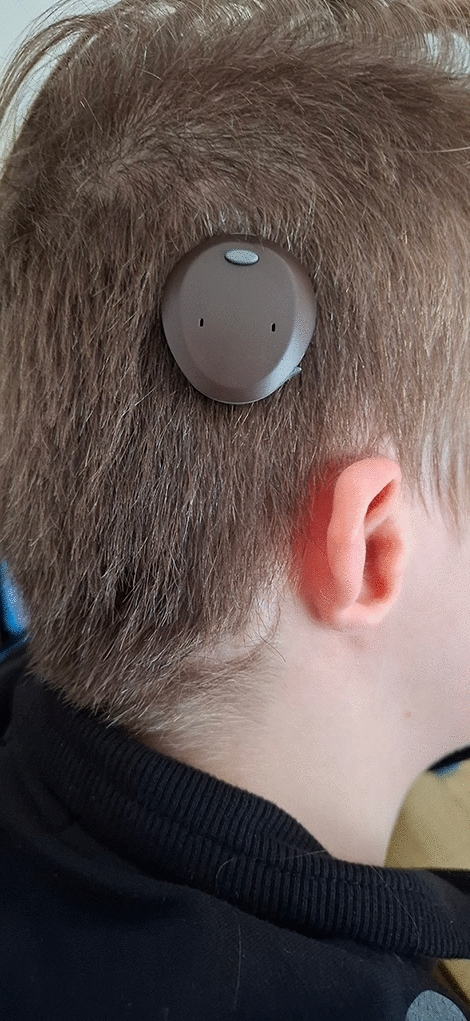
Postoperative appearance and speech processor position

A three layer suture with 3.0 Vicryl for the muscular and subcutaneos layer and Ethilon 4.0 for the skin layer is performed. A light-pressure head bandage is applied for 2 days. Stitches are removed 10 days after the operation and the external sound processor first fitting takes place 4 weeks after surgery (Fig. 5).

## Results

With this surgical technique we implanted 21 patients, 8 female and 13 male between 6 and 83 years old. There have been no postoperative complications regarding skin tension, infection or implant extrusion more than 1 year after the operation. One patient had pain above the implant coil, which was resolved by reducing the magnet strength.

## Discussion

The new system OSI300 is 1.5 and 3 T MR-Conditional. Since this is a very welcomed feature, especially in children, it is to be expected that more MRIs will be performed and tension forces will act on the implant site and potentially become an issue in the future. The skin incision and implant coverage is of great importance. The „old school“ code of practice „no sutures over the implant“, here especially over the actuator which is 6 mm thick, should still be observed.

A two layer dissection modification in OSIA surgery has been described for the minimal invasive surgery (MOSIA) in children by Alnoury et al. [8]. For this technique, the authors prospose a minimal transverse skin incision over the BI300 and align the OSI200 endoscopically to it. The muscle-periosteal flap in our technique is opposing the skin flap to cover the implant and is common practice in cochlear implantation. With our method no skin complications occured.

Regardless of the many variations, the surgical procedure for OSIA [3–8], is reliable, quick and safe, often with less than one hour duration of surgery.

## Summary: 10 key points

1. OSIA surgery is safe, fast and reliable.
2. OSIA3 is MR-Conditional with the magnet in place and needs no head bandage to perform MRI.
3. various incisions have been tried; the most commonly used incision is the J-incision.
4. we recommend no incision over the implant, especially with the OSIA 3.
5. a flat bone region is preferrable for implantation.
6. alternatively, a bed drilling is needed to prevent a bump.
7. repositioning the implant superiorly may be an alternative solution.
8. using the anterior based flap, we did not observe skin issues after surgery.
9. drilling perpendicularly to the bone is mandatory.
10. irrigating prevents bone heating and necrosis.
